# Bromo-protopine, a novel protopine derivative, alleviates tau pathology by activating chaperone-mediated autophagy for Alzheimer’s disease therapy

**DOI:** 10.3389/fmolb.2022.1030534

**Published:** 2022-10-26

**Authors:** Sravan Gopalkrishnashetty Sreenivasmurthy, Ashok Iyaswamy, Senthilkumar Krishnamoorthi, Rambabu N. Reddi, Ananth Kumar Kammala, Karthick Vasudevan, Sanjib Senapati, Zhou Zhu, Cheng-Fu Su, Jia Liu, Xin-Jie Guan, Ka-Kit Chua, King-Ho Cheung, Hubiao Chen, Hong-Jie Zhang, Yuan Zhang, Ju-Xian Song, Siva Sundara Kumar Durairajan, Min Li

**Affiliations:** ^1^ Mr. and Mrs. Ko Chi-Ming Centre for Parkinson’s Disease Research, School of Chinese Medicine, Hong Kong Baptist University, Hong Kong, China; ^2^ Institute for Research and Continuing Education, Hong Kong Baptist University, Shenzhen, China; ^3^ Centre for Trans-disciplinary Research, Department of Pharmacology, Saveetha Dental College and Hospitals, Chennai, India; ^4^ Department of Obstetrics and Gynecology, Division of Basic and Translational Research, The University of Texas Medical Branch, Galveston, United States; ^5^ Department of Biotechnology, REVA University, Bangalore, India; ^6^ Department of Biotechnology, Indian Institute of Technology Madras, Chennai, India; ^7^ School of Chinese Medicine, Hong Kong Baptist University, Hong Kong, China; ^8^ Department of Neurosurgery, Shenzhen Key Laboratory of Neurosurgery, Shenzhen Second People’s Hospital, The First Affiliated Hospital of Shenzhen University, Shenzhen, China; ^9^ Medical College of Acupuncture-Moxibustion and Rehabilitation, Guangzhou University of Chinese Medicine, Guangzhou, China; ^10^ Mycobiology and Neurodegenerative Disease Research Laboratory, Department of Microbiology, Central University of Tamil Nadu, Thiruvarur, India

**Keywords:** Alzheimer’s disease, PRO-Br, chaperone-mediated autophagy, HDAC6, 3xTg-AD, P301S tau

## Abstract

Emerging evidence from Alzheimer’s disease (AD) patients suggests that reducing tau pathology can restore cognitive and memory loss. To reduce tau pathology, it is critical to find brain-permeable tau-degrading small molecules that are safe and effective. HDAC6 inhibition has long been considered a safe and effective therapy for tau pathology. Recently, we identified protopine as a dibenzazecine alkaloid with anti-HDAC6 and anti-AD activities. In this study, we synthesized and tested novel protopine derivatives for their pharmacological action against AD. Among them, bromo-protopine (PRO-Br) demonstrated a two-fold increase in anti-HDAC6 activity and improved anti-tau activities compared to the parent compound in both *in vitro* and *in vivo* AD models. Furthermore, molecular docking results showed that PRO-Br binds to HDAC6, with a ∆G value of −8.4 kcal/mol and an IC_50_ value of 1.51 µM. In neuronal cell lines, PRO-Br reduced pathological tau by inducing chaperone-mediated autophagy (CMA). In 3xTg-AD and P301S tau mice models, PRO-Br specifically decreased the pathogenic hyperphosphorylated tau clumps and led to the restoration of memory functions. In addition, PRO-Br treatment promoted the clearance of pathogenic tau by enhancing the expression of molecular chaperones (HSC70) and lysosomal markers (LAMP2A) *via* CMA in AD models. Our data strongly suggest that administration of the brain-permeable protopine derivative PRO-Br, could be a viable anti-tau therapeutic strategy for AD.

## Graphical Abstract

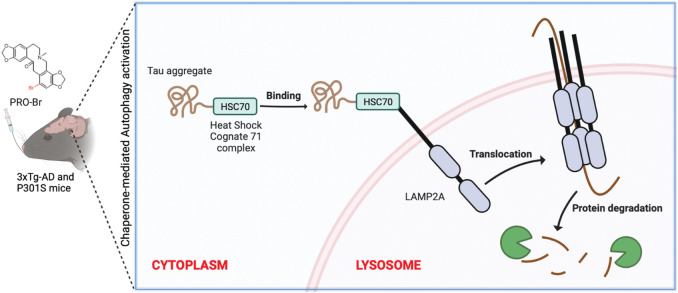

## Introduction

A growing body of evidence suggests that the microtubule-binding protein tau is the primary hallmark of tau pathology in various neural conditions, including Alzheimer’s disease (AD) (van der Kant et al., 2020). Several post-translational changes can cause normal tau protein to be converted into pathogenic tau aggregates (M. Kolarova et al., 2012). These modifications are hyperphosphorylation, acetylation, ubiquitination, and protein truncation. According to recent research, the longest tau isoform contains 80 serine and threonine amino acids; these are potential targets for aberrant phosphorylation (Sergeant et al., 2005). Recent study findings indicate that targeting tau pathology may succeed where Aβ pathology-reducing medications have failed (Cummings et al., 2020); (Arnsten et al., 2020). For this reason, we focus on reducing early tau pathogenesis as a potentially effective strategy for treating AD.

Proteolytic processes implicated in tau breakdown include macroautophagy (also known as autophagy) and the ubiquitin-proteasomal system (UPS), both of which are essential in maintaining cellular homeostasis (Lee et al., 2013); (Scrivo et al., 2018); (Guan et al., 2022). Previous research from our group and others have revealed that autophagy and UPS play a role in tau clearance in multiple AD models (Iyaswamy et al., 2021); (Sreenivasmurthy et al., 2022). Furthermore, multiple investigations have shown that both degradation processes are dysregulated in AD (Kocaturk and Gozuacik, 2018). Therefore, addressing other tau-degradation mechanisms seems a viable strategy for treating AD.

Chaperone-mediated autophagy (CMA) was one of the earliest cellular pathways studied for the clearance of protein aggregates (Kaushik and Cuervo, 2018). In AD, CMA dysfunction promotes the build-up of harmful proteins (Cuervo et al., 2004). CMA does not require the formation of double-membrane structures to engulf cytosolic components; instead, specialized heat shock proteins, such as HSC70, can identify the KFERQ pattern found on cargo proteins (Kaushik and Cuervo, 2018). Multiple similar motifs are observed in hyperphosphorylated tau protein. HSC70 recognizes these motifs, and uses them to form a complex (Young et al., 2016) which attaches to lysosomal surface proteins such as lysosomal-associated membrane proteins 2A (LAMP2A) and clears tau inside the lysosomal lumen (Auzmendi-Iriarte and Matheu, 2021).

Previous research on post-mortem brain tissues from AD patients found a 90% increase in the expression of histone deacetylase 6 (HDAC6) (Soeda and Takashima, 2020); (Simões-Pires et al., 2013). HDAC6 has been found in immunofluorescence and co-immunoprecipitation tests to interact with aberrant tau and produce tau aggregates in AD models. HDAC6 also has various other activities, including interacting with ubiquitinated misfolded proteins like tau and forming aggresomes (Simões-Pires et al., 2013). Inhibiting HDAC6 activity reduces tau aggregation and has been proven to protect against cognitive dysfunctions in AD models (Simões-Pires et al., 2013). One of the most recent and promising drug development methodologies for AD therapy is the use of HDAC6 inhibitors (HDAC6is) to target tau breakdown. Furthermore, RNA interference-mediated HDAC6 knockdown enhances the expression of HSC70 and LAMP2A in PC12 cells (Su et al., 2016). In Parkinson’s disease rat model, administration of tubastatin A, a HDAC6 selective inhibitor, promoted the clearance of α-synuclein, and protected the dopaminergic neurons, *via* activating CMA (Francelle et al., 2020). Therefore, HDAC6 seems a valid pharmacological target for addressing tau pathology in AD (Falkenberg and Johnstone, 2014); (Passaro et al., 2021). However, many HDAC6 inhibitors have recently been reported to exhibit qualities unacceptable for a drug, such as high lipophilicity and profound toxicity (Diedrich et al., 2016). Therefore, finding novel HDAC6 inhibitors that control HDAC6 activity, clears tau aggregates *via* CMA, is non-toxic, and is bioavailable is the goal.

Protopine (PRO) is a dibenzazecine alkaloid isolated from *Corydali*s and *Fumaria* species. According to our previous study, PRO is an effective anti-tau agent that enhances memory functions in AD models (Sreenivasmurthy et al., 2022). Other researchers have found that PRO possesses anti-inflammatory, anti-oxidative, anti-nociceptive, anti-acetylcholinesterase, and anti-depression activities in mice. However, PRO’s oral bioavailability remains low. In an attempt to boost its biological activity, we brominated PRO, producing PRO-Br, and tested its anti-tau activity in AD models. Hence, we hypothesized that PRO-Br can significantly pass through the BBB, and mitigate memory dysfunction in AD models. Therefore by using HDAC6 profiling and immunofluorescence assays, we aimed to determine that PRO-Br mediated the degradation of pathological tau. Furthermore to check the anti-AD efficacy of PRO-Br, we used both 3xTg-AD and P301S tau mice.

## Materials and methods

### Animals and PRO-Br treatment

To determine the amount of PRO-Br in the brain, a pharmacokinetic study was performed employing 3-month-old female ICR mice (n = 6, 30 ± 2 g). PRO-Br (10 mg/kg b. wt) was injected orally. Brain tissues and blood samples (intracardial) were collected at various time-points (0, 15, 30, 45, 60, 75, 120, 240, 360, 480 min). Brain tissues and plasma samples were kept at −80°C until analysis. On the day of analysing the samples, the brain tissues were thawed on ice and half brain tissue was taken for analysis. To 200 mg of tissue, 4 times the volume of methanol (100%) was added, and homogenized until a clear solution was observed. The samples were centrifuged at 15,000 RPM for 10 min at 4°C. The supernatant was separated and analyzed using LC-MS/MS.

Female homozygous 3xTg-AD mice carrying transgenes for *APP* (Swedish KM670/671Nl), *MAPT* (tauP301L) and *Psen1* (M146V) were procured from Jackson’s Laboratory (# 004807). The transgenic mice colonies were bred and maintained in the HKBU animal house at 23 ± 2 °C, 60 ± 15% relative humidity with regular access to food and water. Seven-month-old 3xTg-AD mice were randomly segregated into three treatment groups: Tg-Vehicle, 1 mg/kg, and 2.5 mg/kg PRO-Br. PRO-Br was administered orally for 8 months (n = 8). The bodyweight of mice from all the groups was recorded once every week.

Female homozygous P301S mice bearing the transgene for *MAPT* (P301S), were generous gifts from Michael Goedert. P301S-tau mice (1.5-month-old, n = 8) were administered with PRO-Br daily for 2.5 months. The mice models employed in the study were maintained under 12 h light-dark cycles and were provided *ad libitum* access to chow and water.

Animal breeding, maintenance, PRO-Br treatment and behavior assays were approved by the HKBU and the Committee on the Use of Live Animals in Teaching and Research (CULATR #4414) at the University of Hong Kong. The animal license reference number is (18–46) in DH/SHS/8/2/6 Pt.2.

### Morris water maze test

To determine the memory functions of experimental mice, the Morris water maze test was performed according to the procedure described previously (Iyaswamy et al., 2020). Mice training preliminary to the probe trail was performed in two phases. The first phase, known as the visible-platform training, comprised four trials, for 1 day. In each trial, a platform was placed in the tank, 1 cm below the surface of the (clear) water. The mouse was introduced, and the time required for the mouse to find the platform was recorded. The position of the platform was changed for every trial. The mice that performed well during the visible-platform training were taken for the next phase. In the second training phase, non-toxic white paint was mixed into the water, and the platform was placed at a constant location in the tank at the same depth, but now hidden from sight due to the opaque water. Mice from all the groups were trained to find the hidden platform for six consecutive days (4 trials daily, 1 min per trial). On the seventh day, a probe trial was conducted to assess memory retention. The distance travelled (cm) to reach the platform in the target quadrant was observed and recorded by a video tracking method (Ethovision, Noldus Information Technology, Version 3).

### Rotarod test

Motor behavior was assessed by the rotarod test (Harvard apparatus, SeDaCom v2.0.000) and was performed according to the procedure described previously (Song et al., 2020). The study mice were brought into the experimental room 30 min before the test. The training was conducted with a constant speed of 4, 8, and 12 rpm on days 1, 2, and 3, respectively. Three trials were conducted each day and the performance was monitored. On the fourth day, an acceleration mode (4–40 rpm in 5 min) was introduced, and the time spent (s) by each mouse on the rod was recorded by the instrument connected to a computer.

### Contextual fear conditioning test

After treating P301S mice with PRO-Br, fear memory consolidation was evaluated by the contextual fear conditioning (CFC) test. The experimental set-up consisted of two individual sound-proof chambers, each having an electric grid on the surface. Inside each chamber, a speaker was attached to introduce a cue tone. The movement of the mice was observed and recorded by ANY maze tracking software on a laptop. The experiment was performed with a continuous white noise (40 units) and white light (40 lux). On the first day of the training session, mice were introduced into the chamber and permitted to explore for 2.5 min. Next, each mouse received three consecutive foot shock cycles; each started with a 28 s cue tone (1,500 Hz) and finished with a 2 s foot shock (30.0 mA). On the second day, the mice were allowed to explore the grid for 3 min, after which they received a cue tone for 30 s without foot shock, and the freezing time was recorded.

### Immunohistochemistry (IHC)

Brain tissues from experimental mice were carefully isolated, washed with 1X PBS, and fixed with 4% paraformaldehyde. Post-fixation for 48 h, brain tissues were transferred to 30% sucrose solution for cryopreservation. Tissue sections (30 μm) were obtained using a cryotome, maintained at a constant -30°C. Permeabilization was performed with 0.4% triton-X-100 for 10 min. For tau immunofluorescence, PHF1 primary antibody was added, and sections were kept overnight at four°C. The following day, an appropriate secondary antibody was added at RT for 1.5 h. DAPI was used as a nuclear stain for 5 min. For DAB staining, AT8 primary antibody was added to the sections overnight. The next day, an appropriate secondary antibody was added for 1.5 h. Staining was performed by employing a Vectastain-ELITE ABC-HRP kit according to the manufacturer’s procedure. The stained sections were carefully arranged on glass slides and mounted with an appropriate mounting medium under a coverslip. Images of cortical/hippocampal regions were taken with an Eclipse 80i fluorescence microscope (Nikon Instruments Inc.). The threshold was adjusted to remove the background staining. The captured images were quantified using ImageJ (NIH) software.

### Golgi staining of dendritic spines

FD Rapid Golgi Stain Kit was employed to determine the dendritic spine density in the 3xTg-AD mice brains and was performed according to procedure described previously (Iyaswamy et al., 2022). Briefly, mice were perfused using 4% PFA, and the brains were dissected carefully to avoid damage. Next, the brain samples were frozen by dipping in isopentane solution (on dry ice). The frozen brains were sectioned at 150 μm thickness using a Shandon Cryotome SME Cryostat (Ramsey, MN, United States). The tissue slices were carefully mounted onto glass slides and stained with the kit reagents in accordance with the manufacturer’s procedure. The dendritic images were acquired by Leica confocal microscope. NIH ImageJ Sholl Analysis software was used for measuring the spine head length, breadth, and linear density of the cortex and the hippocampus. The criteria for measuring the dendritic spine morphology and spine density were set up following the previously described protocol (Song et al., 2014). For analysis of dendritic spine density, the spine lengths of 0.2–2 μm from each mouse (n = 8 per group) were used. To analyze spine-morphology, spines of up to 3.5 μm in length and 1.5 μm in width were considered.

### Mass spectrometry analysis

Quantification of PRO-Br in mice brains was performed by Agilent 6410 LC-ESI-Triple-Quad/TOF. The mobile phase (A) consisted of water with 0.1% formic acid, and the mobile phase (B) consisted of acetonitrile with 0.1% formic acid (B). C18 column was used to elute the desired solutes, based on the protocol: 0–5 min, 23–50% B; 5–15 min, 50–60% B; 15–15.01 min, 60–95% B; 15.01–22 min, 95% B. The ions of PRO-Br were detected in positive ionization mode. Multiple reaction monitoring (MRM) mode was used to monitor the analytes of PRO-Br.

### Synthesis of PRO-Br

Protopine (Cat. No. APC-458) was procured from Atkin Chemicals Inc. and the purity of the compound is 98% min according to the manufacturer. Protopine solution (353 mg, 1 mmol) in CH_3_CN: H_2_O (3:1, 6 ml) was constantly stirred, to which N-bromo succinimide (213 mg, 1.2 mmol) was added at 0°C. Next, the reaction mix was continuously stirred for 2 h at RT. After completion of the reaction (as monitored by TLC), CH_3_CN was concentrated *in vacuo*, and the aqueous layer was extracted with EtOAc (3 × 3 ml). The combined organic layer was concentrated *in vacuo*, and the crude product was purified by column chromatography using MeOH: CH_2_Cl_2_ (2:8) as eluent to give pure PRO-Br (335 mg) (yield = 77%).

### *In silico* docking and molecular dynamic simulation of PRO-Br with HDAC6

A molecular docking approach was used to determine the binding affinity and critical interaction between target protein and compounds. The 3D structures of HDAC6 (5EDU) were retrieved from the RCSB PDB database (https://www.rcsb.org/). The 3D structures of compounds was sketched using Chemsketch 2021.2.1 (https://www.acdlabs.com/resources/freeware/chemsketch/) and converted to PDB structures using PyMOL v1.8 software (https://pymol.org/). AutoDockTools (version 1.5.6) was utilized for the addition of hydrogen atoms and for computing the Kollman charges (Morris et al., 2009). Binding site information was retrieved from literature and the LPC CSU Server (https://oca.weizmann.ac.il/oca-bin/lpccsu) (Hai and Christianson, 2016). The PDB format of both target protein and compound was converted to pdbqt format. AutoDock Vina (Trott and Olson, 2010) was used to analyze molecular docking between the target protein and compounds. Repeated docking was carried out to ensure the consistency of molecular docking results and to determine the best binding pose(s). The key intermolecular interactions and the binding mode were depicted using BIOVIA Discovery Studio v.2021 and Chimera v.1.16 (TE Pettersen EF et al., 2004).

### Reagents and antibodies

Cell culture requirements: DMEM/F12 (11320082), DMEM (11965084), Trypsin-EDTA (15400054), glutamine (25030081), Blasticidin (A1113903), and Fetal bovine serum (16000044), were procured from ThermoFisher Scientific. Protopine (APC-458, purity is 98%min by HPLC) was purchased from Aktin Chemicals Inc. HDAC6 Fluorometric Drug Discovery Kit (BML-AK516) was purchased from Enzo Life Sciences. Western blotting requirements: PVDF membrane (GE10600021), ECL chemiluminescence detection kits (32106), phosphatase inhibitor tablets (A32957), and protease inhibitor tablets (4693159001, Roche), were procured from ThermoFisher Scientific. Anti-mouse IgG (115–035–003) and anti-rabbit IgG (111–035–144) secondary antibodies were procured from Jackson ImmunoResearch Laboratories.

### Cell culture

SHSY5Y cells stably-expressing mutant tau (SHSYP301L) were cultured in DMEM/F12 + 15% FBS, 3 μg/ml Blasticidin S HCl, and 1X PSN. N2A mouse neuroblastoma and mouse hippocampal neuronal (HT22) cells were cultured in DMEM+10% FBS, and 1X PSN. Sterile complete medium containing PRO-Br was added to the cells, and they were then incubated for 24 h at an optimum temperature of 37°C in a sterile 5% CO_2_ incubator.

### Tau extraction

Brain tissues of 3xTg-AD and P301S mice were separated, sagittally, into equal halves. One-half was lysed with tissue lysis buffer augmented with phosphatase and protease inhibitor cocktail tablets, while the other half was used for immunohistochemical analysis. Brain tissues were homogenized on ice using an Ultra Turrax tissue homogenizer. Brain tissue homogenate was subjected to sarkosyl differential centrifugation with 1% sarkosyl to separate the non-pathogenic soluble and pathogenic insoluble tau fractions. Protein lysates were evaluated for changes in levels of phospho epitopes of tau (AT8, CP13, PHF-1, MC-1, and ALZ50), and for total htau (HT7) using Western blot.

### Western blot

After PRO-Br treatment, cells were solubilised using cold RIPA lysis buffer (9803, Life Technologies), containing protease and phosphatase inhibitor tablets. Cells were sonicated and centrifuged at 14000 rpm for 15 min. Total protein concentration was determined by BCA protein assay, and the samples were separated on SDS-PAGE and transferred to PVDF membranes. The membranes were blocked, washed, and incubated with primary antibodies (Table 1), and incubated at 4°C, overnight. The membranes were further incubated with the required dilutions of secondary antibody for 1 h, at RT. The chemiluminescent signals were captured using x-ray film in a darkroom, with an appropriate chemiluminescent substrate kit (ThermoFisher Scientific, United States). Films were scanned, and the relative intensities of the signals were analyzed using ImageJ software.

**TABLE 1:** Details of the primary antibodies used.

Antibody	Source/Company	Catalog No.	Assays and Dilution used
ALZ50	Prof. Peter Davies, Albert Einstein College of Medicine, NY, United States	—	WB 1:1,000
AT8	Prof. Peter Davies, Albert Einstein College of Medicine, NY, United States	—	WB 1:1,000, IHC 1:500
CP13	Prof. Peter Davies, Albert Einstein College of Medicine, NY, United States	—	WB 1:1,000
MCI	Prof. Peter Davies, Albert Einstein College of Medicine, NY, United States	—	WB 1:1,000
PHFI	Prof. Peter Davies, Albert Einstein College of Medicine, NY, United States	—	WB 1:1,000, IHC 1:500
HT7	Thermoscientific, Waltham, MA USA′	# MN1000	WB 1:1,000
TAU5	Thermoscientific, Waltham, MA. United States	# AFIB0042	WB 1:1,000
Acetylated a-tubulin (Lys40)	Thermoscientific, Waltham, MA′ UT	# 32–2700	WB 1:20000
α-tubulin	Abcam, Waltham, MA, United States	# ab18251	WB 1:20000
ACTB (β-actin)	Santa Quz Biotechnology, Inc., Dallas, Texas, United States	# sc-47778	WB 1:10000
HSP70	Enzo Biochem, Farmingdale, NY,United States	# ADI-SPA-810- F	WB 1:1,000
HSC70	Enzo Biochem, Farmingdale, NY,United States	# AIX-804–067–8050	WB 1:1,000
HSP90	Enzo Biochem, Farmingdak, NY, United States	# ADI-SPA-846- D	WB 1:1,000
AcetYlated HSP9° 0 (294)	Rockland hzmamochemicals, LthrlidC, PA	# 600401–981	WB 1:500
HDAC6	Cell signaling, Danvers, MA, United States	# 7612	WB 1:1,000

### Immunocytochemistry (ICC)

HT22 cells were cultured in sterile 24-well culture dishes containing 14 mm cover-glass. Cells were treated with PRO-Br (12.5 and 25 μM), and DMSO (0.1%), for 24 h. Cells were fixed with paraformaldehyde (4%), permeabilized with triton-X-100 (0.1%), and blocked with BSA (2%). LAMP2A antibody (1:500), and HSC70 antibody (1:500) was added to the processed cells and incubated overnight at four°C. Alexa Fluor^®^ 488 (green) and Alexa Fluor^®^ 594 (red) secondary antibodies (1:400) were added and incubated for 1.5 h at room temperature. FluorSave reagent (Merck Millipore, United States) was added, then cover-slipped, and images were captured by a Leica confocal imaging system.

### HDAC6 deacetylase assay

Inhibition of HDAC6 activity was performed in accordance with the manufacturer’s protocol. Briefly, PRO-Br at various concentrations in HDAC Assay buffer II were prepared. The appropriate substrate solution provided in the kit was added, and the reaction plate was incubated at 37°C. The reaction was stopped by the addition of Developer II, and the fluorometric reading was measured at 440 nm using a fluorometer (Wallac EnVision).

### Statistical analysis

Data was quantified with one-way and two-way analysis of variance (ANOVA). Data were represented as the mean ± S.E.M. Bar diagrams were generated to evaluate the normality of the data. For normally distributed data, statistical analysis was done using one-way ANOVA, followed by a post hoc comparison of the means using Bonferroni’s or Dunnett’s methods. Probability value of *p* < 0.05 was considered significant. Statistical significance and graphical representations were performed by GraphPad Prism six Software.

## Results

### Structural modification of PRO

**FIGURE 1:**
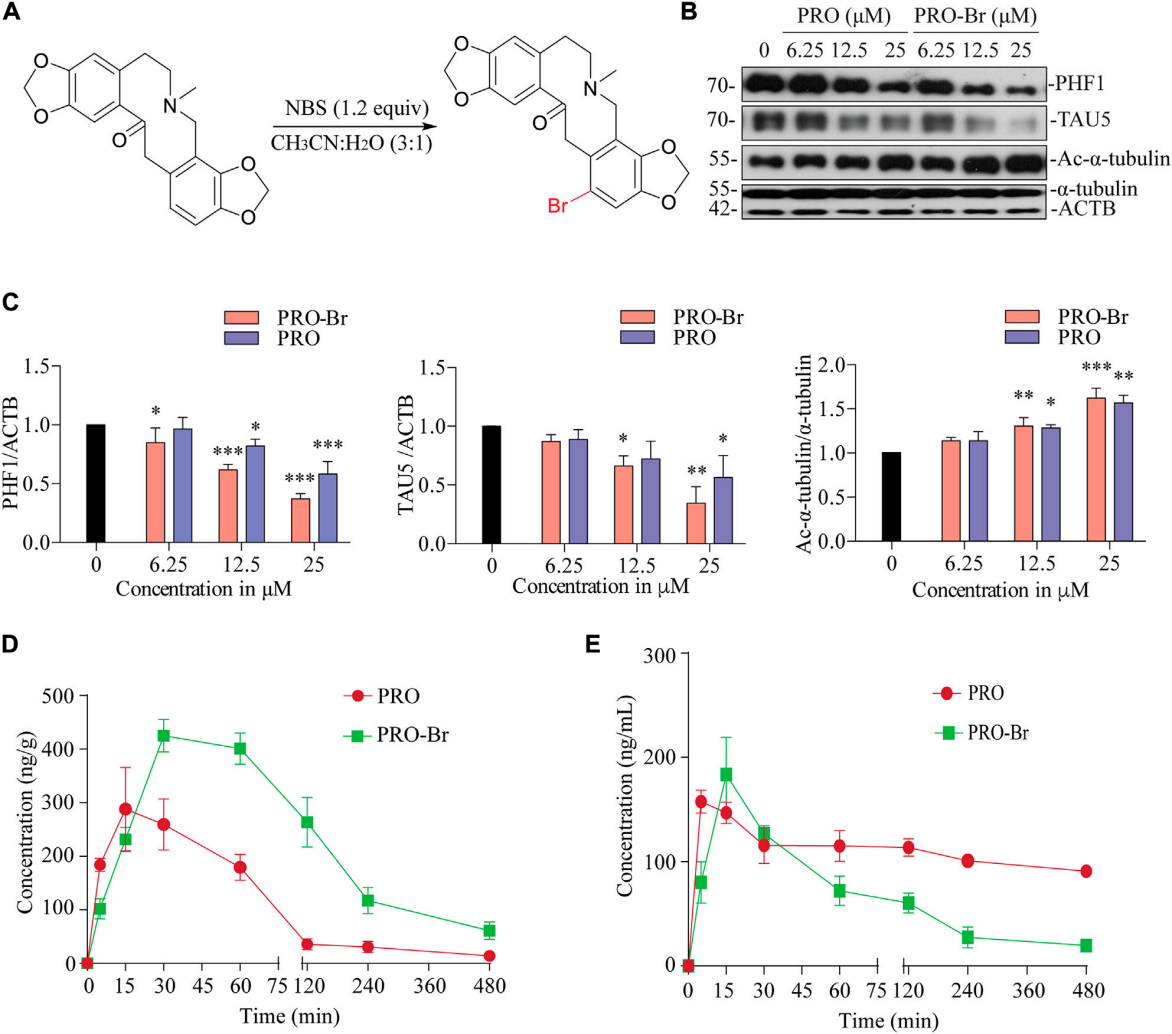
PRO-Br inhibits HDAC6 and promotes tau degradation **(A)** Scheme showing synthesis of PRO-Br from PRO. In SHSYP301L cells, **(B)** PRO-Br exhibited a 2-fold increase in potency compared to PRO for reducing HDAC6 deacetylase activity and tau accumulation **(C)** Data are presented as mean ± SEM of three replicates in a representative experiment for PHF1, TAU5 and Ac-α-tubulin. Pharmacokinetic profiles of PRO-Br (10 mg/kg) in **(D)** brain and **(E)** plasma.

Initially, we modified the structure of PRO at the 10-membered ring, which is adjacent to the carbonyl group. Alkylation was performed with various alkyl (methyl, allyl and benzyl) halides. With methyl iodide, PRO gave the corresponding α-methylated product (3a), while allyl bromide and benzyl bromide gave dialkylated products (3b and 3c, respectively; Supplementary Figure S2A). The structure of PRO was modified as mentioned above to obtain nine derivatives of the parent compound. Firstly, a cytotoxicity assay (MTT) was performed *in vitro* to determine the optimum concentration for downstream analysis of the synthesized compounds (data not shown). Next, the tau-reducing efficacies for all the synthesized PRO derivatives were determined in SHSYP301L tau-overexpressing cells. Surprisingly, none of these protopine derivatives could reduce pathological tau/PHF1 *in vitro* (data not shown). These results suggest that the modification at the 10-membered ring of PRO decreased its activity and we concluded that the 10-membered ring is important for PRO’s biological activity. Hence, the C12 position of PRO was chosen for the synthesis of Protopine-Br (PRO-Br). We performed the bromination of PRO with N-bromosuccinimide (NBS) (Figure 1A) and obtained the corresponding PRO-Br derivative with a yield of 77%. In our preliminary screening, PRO-Br demonstrated good tau/PHF1-reducing activity, and enhanced the acetylation of α-tubulin compared to its parent compound, PRO (Figures 1B,C). Therefore, we chose to explore the neuroprotective efficacy of PRO-Br and its downstream mechanism in AD models.

The physicochemical characteristics of PRO-Br were evaluated by NMR (Supplementary Figures S3A, B), and LC-MS/MS (Supplementary Figure S3C). From this, the structure of PRO-Br was determined to be a derivative of PRO bearing a bromo moiety at the C12 position (Supplementary Figure S3D).

### PRO-Br improves the cognitive and memory functions in AD mice models

One of the most important criteria for a drug to effectively treat CNS-related diseases is its brain permeability. Hence, the pharmacokinetics of PRO-Br specifically in the brain was assessed in wild-type female ICR mice. After oral administration of PRO-Br (10 mg/kg), brain and plasma samples were analysed at indicated time-points. The maximum concentration (C_max_) attained was found to be 425.22 ng/g in the brain at a maximum time (T_max_) of 30 min. PRO-Br was retained in the brain for 4.2 h. The C_max_ of PRO-Br in the plasma was observed to be 91.86 ng/ml at a T_max_ of 15 min. The brain/plasma ratio of PRO-Br was calculated to be 8.08. According to the results, the brain penetrating efficacy of PRO-Br was superior compared to its parent compound protopine (PRO). Additionally, the clearance rate of PRO-Br in the plasma (7.4 ng/ml) was observed to be better than PRO (2.5 ng/ml) (Figures 1D,E, Supplementary Figures S1A, B).

**FIGURE 2:**
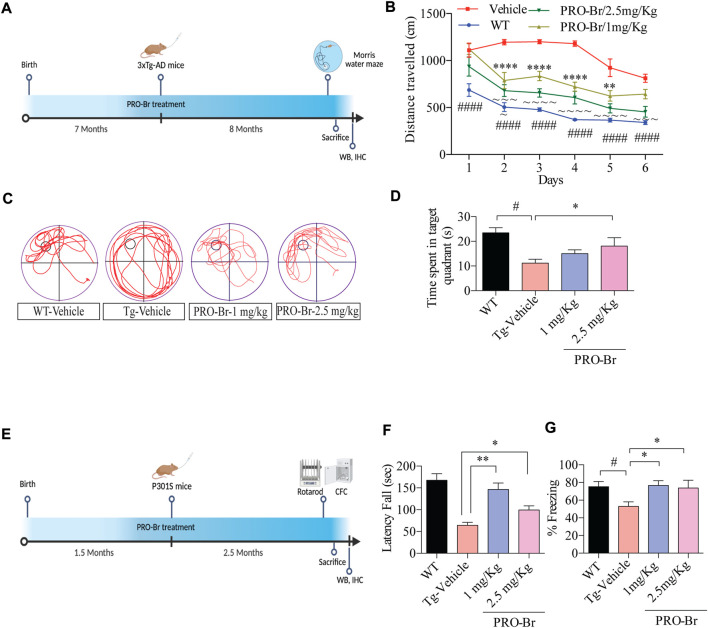
PRO-Br alleviates cognitive impairment in AD mice models **(A)** Schematic timeline of PRO-Br treatment and behaviour schedule in 3xTg-AD mice model. **(B)** PRO-Br improved memory function according to the Morris water maze test for WT-Vehicle, Tg-Vehicle-, and PRO-Br-treated groups (1 and 2.5 mg/kg) **(C)** Representative images of path-lengths traversed by the mice of all groups to reach the target quadrant. **(D)** Probe test shows the time spent by animals in the target quadrant **(E)** Schematic timeline of PRO-Br treatment and behaviour schedule in P301S mice model. **(F)** PRO-Br treatment increased the motor functions in the rota-rod assay and **(G)** increased the freezing time and improved the hippocampal memory functions in CFC assay in the P301S mice model.

Next, the effect of PRO-Br on memory dysfunctions was assessed in triple transgenic mice (3xTg-AD). These mice carry APP (Swedish mutation) and tau (P301L mutation), well-known for causing extracellular plaques and intracellular NTFs, respectively, and cognitive dysfunctions. Oral administration of PRO-Br significantly reversed the learning and memory impairment in 3xTg-AD mice (Figures 2A–D). Earlier reports have demonstrated a dysfunctional spine density in the brain regions of AD mice models (Dorostkar et al., 2015); (Maiti et al., 2021). Reduction in spine density leads to an imbalance in synapse formation, thereby causing improper cell-to-cell communications. Therefore, we studied the impact of PRO-Br administration on spine density. PRO-Br significantly enhanced the dendritic spine structures (thin, stubby and mushroom-like) in the hippocampus of 3xTg-AD mice, indicating an improvement in spine maturation. PRO-Br-mediated improvement in spine density could explain the enhanced cognitive and memory functions in AD mice treated with PRO-Br (Supplementary Figures S4A, B).

The tau-reducing efficacy of PRO-Br was further evaluated in the P301S mutant tau overexpressed mice model. At 4 months, P301S tau mice suffered from paralysis, which severely affected its motor functions. Therefore, for this study we employed 1.5-month-old mice, which were subsequently orally treated with PRO-Br for 2.5 months. Recent reports have demonstrated that loss of motor functions, a non-cognitive symptom, has also been observed in AD patients (Buchman and Bennett, 2011). Furthermore, other studies have shown that rotarod and contextual fear conditioning assays evaluated the learning and memory functions in tau-overexpressed transgenic model mice (Takeuchi et al., 2011). Therefore, upon evaluating the rotarod assay results, we observed that in the PRO-Br-treated group, the latency of falling was significantly enhanced in comparison to the Tg-Vehicle group (Figures 2E,F). Additionally, associative learning and memory functions were assessed by contextual fear conditioning (CFC) assay. Compared to the WT mice, P301S mice showed a significant decrease in freezing time. However, the P301S mice that were treated with PRO-Br showed significant increase in freezing percentage, and rescued memory dysfunction (Figure 2G). Therefore, our findings indicate that PRO-Br decreased sensorimotor dysfunctions, and enhanced learning and memory functions in the P301S AD model.

### PRO-Br can promote the clearance of pathological tau in AD models

**FIGURE 3:**
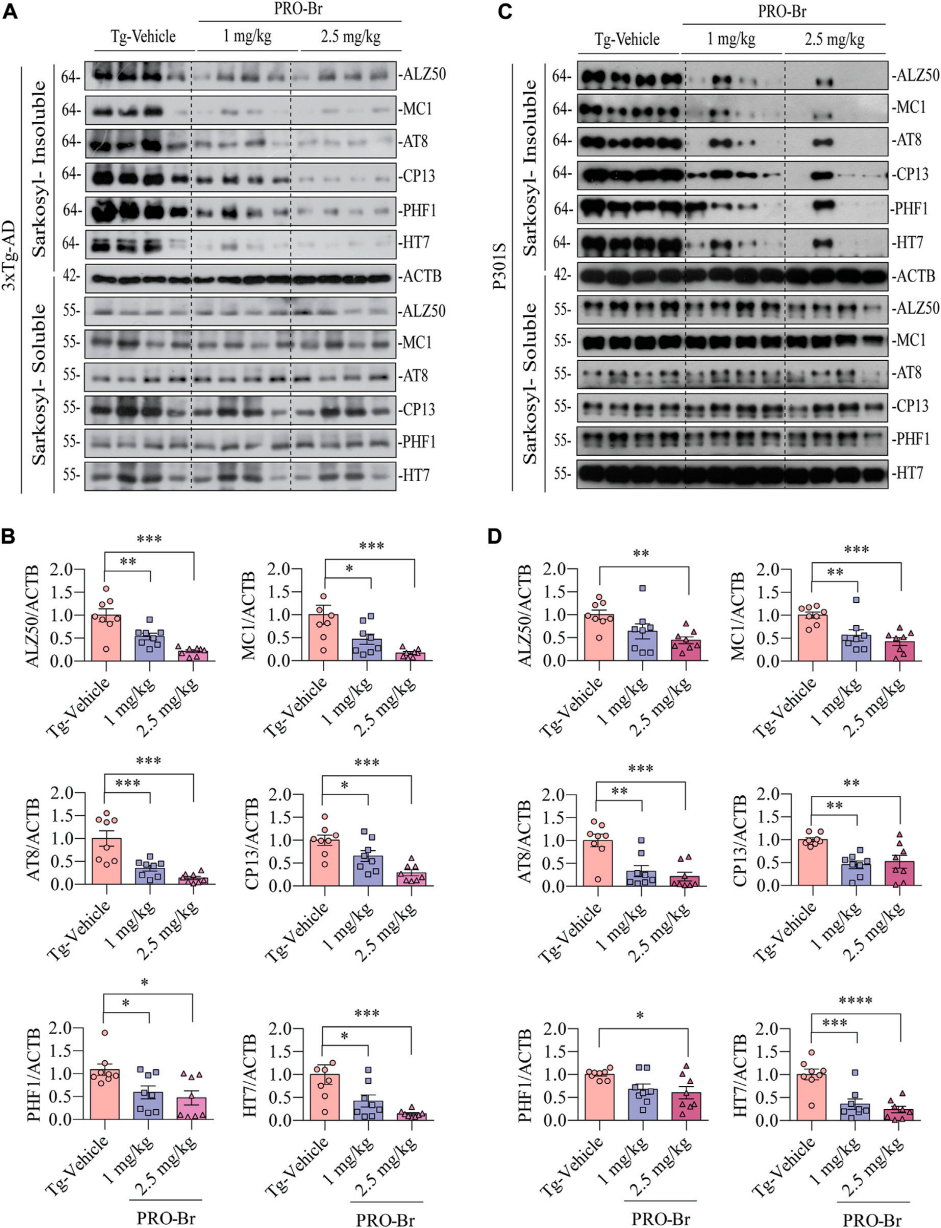
PRO-Br reduced the insoluble phospho-tau in AD mice. PRO-Br specifically decreased sarkosyl-insoluble tau in **(A-B)** 3xTg-AD and **(C-D)** P301S mice models. Data is represented as mean ± SEM. N = 8 in each group, the statistical significance are *p < 0.05, **p < 0.01, ***p < 0.001 (Tg-PRO-Br/PRO V. Tg-Vehicle).

**FIGURE 4:**
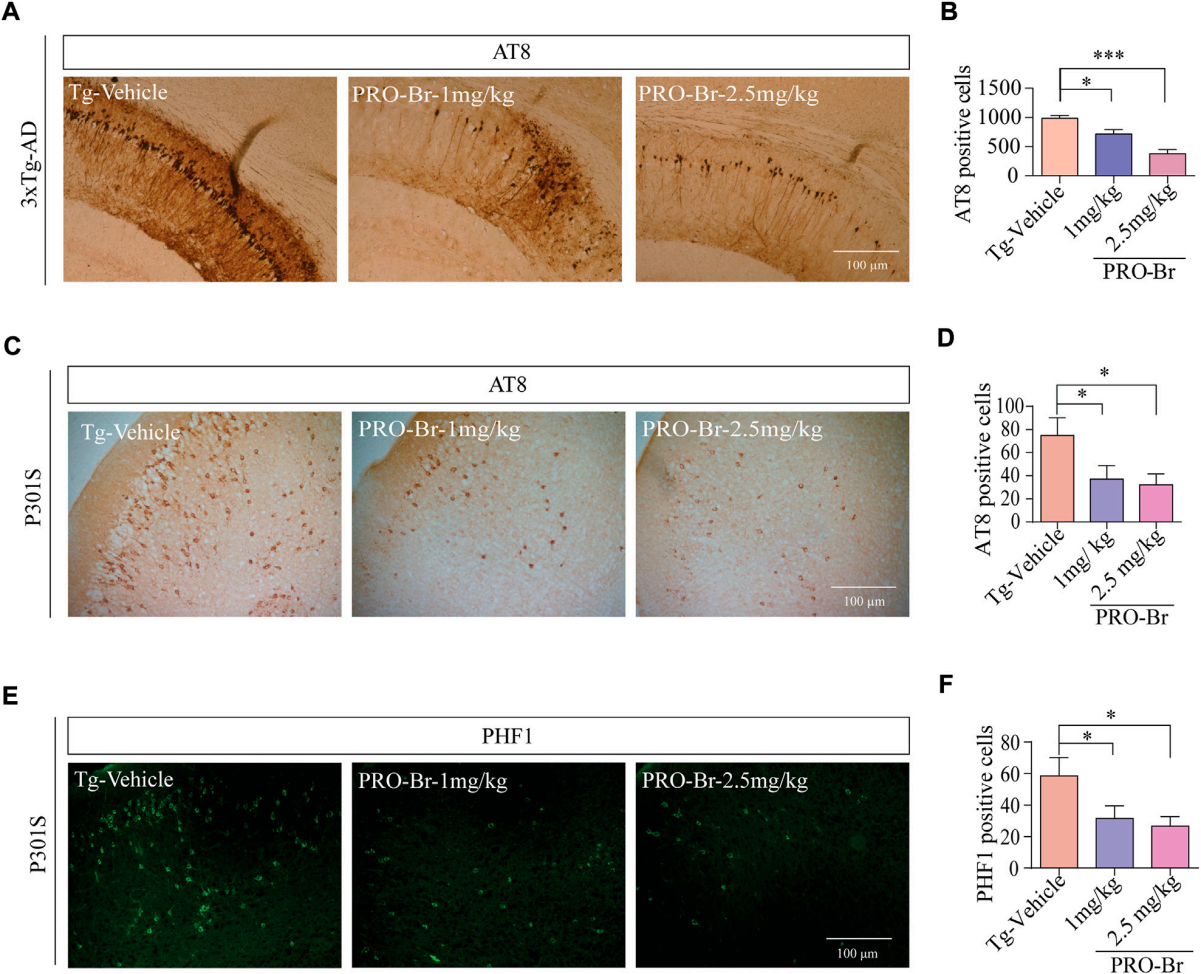
Immunohistochemical analysis of brain tissues of PRO-Br-treated AD mice. Images of brain tissue slices showing that PRO-Br reduces **(A-B)** AT8-positive cells in 3xTg-AD mice and also reduced AT8-and PHF1-positive cells in **(C-F)** P301S tau mice dose-dependently. Data was quantified using ImageJ software, *p < 0.05 and ***p < 0.001.

AD progression is associated with abnormal Aβ plaques extracellularly and tau tangles intracellularly. Previous studies have demonstrated that intracellular hyperphosphorylated tau tangles directly correlate with neurotoxicity in AD (Augustinack et al., 2002). Sarkosyl-mediated extraction is a well characterized process for isolating insoluble tau from the brains of AD mice. Therefore, we used this method to evaluate the levels of non-pathological and pathological tau in 3xTg-AD and P301S tau mice models of AD. Since phosphorylation at various sites on tau creates pathological tau, we investigated the PRO-Br-mediated changes in expression levels of several phospho-tau epitopes (ALZ50, MC1, AT8, CP13, PHF1, and HT7). Immunoblotting results from both AD mice samples demonstrated that PRO-Br specifically reduced the expression of sarkosyl-insoluble phospho-tau variants in a dose-dependent manner (Figures 3A–D). Histochemical analysis of brain slices demonstrated that PRO-Br at doses of 1.0 and 2.5 mg/kg significantly reduced AT8-positive cell count in the cortico-hippocampal regions in 3xTg-AD mice (Figures 4A,B), and AT8-, and PHF1-positive cell counts in P301S tau mice (Figures 4C–F).

### PRO-Br binds to and inhibits the activity of HDAC6

**FIGURE 5:**
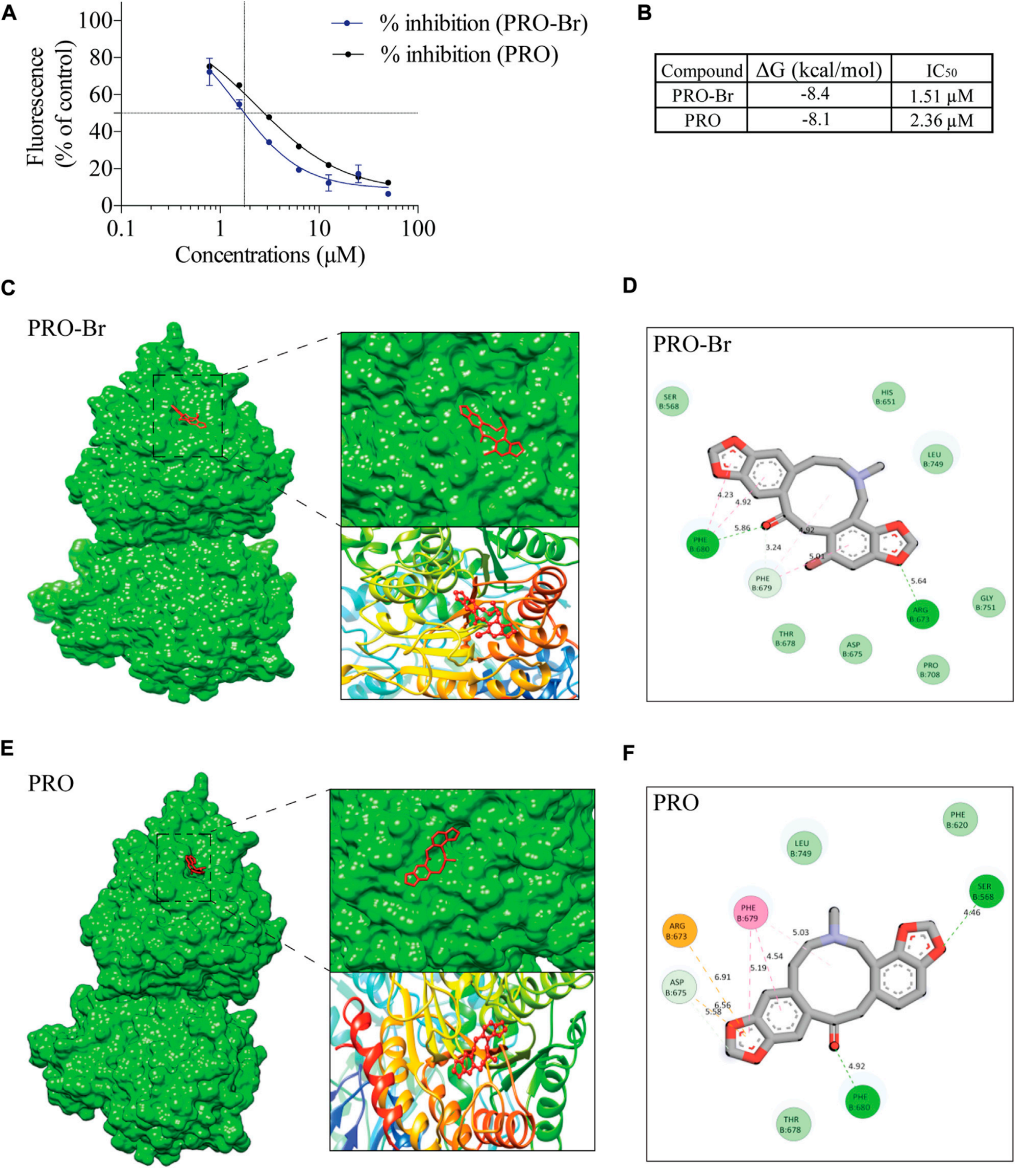
PRO-Br specifically binds to HDAC6 **(A)** PRO-Br/PRO can dose-dependently inhibit the activity of HDAC6. **(B)** Table shows the 50% inhibitory concentration (IC_50_) values of PRO-Br and PRO. Estimated free energies values of PRO-Br/PRO are as shown in table. **(C–F)** Visualization of the binding and key interacting amino acids between PRO-Br/PRO and HDAC6.

HDAC6 is a cytoplasmic deacetylase that targets key proteins such as α-tubulin and HSP90. Many studies have demonstrated that HDAC6 plays a crucial role in the accumulation of hyperphosphorylated tau aggregates in AD. Therefore, inhibition of HDAC6 activity could be a promising therapeutic strategy for AD. Hence, to determine whether PRO-Br affects HDAC6 activity, we conducted fluorometric assay with pure HDAC6 protein. Our results demonstrate that PRO-Br can reduce HDAC6 activity in a concentration-dependent manner (Figure 5A). The calculated 50% inhibitory concentration of PRO-Br was found to be 1.51 µM (Figure 5B). To determine the binding of PRO/PRO-Br to HDAC6, we used molecular docking, and the binding affinity was determined from the binding free energies (Kcal/mol). From the docking study, the binding free energy of PRO-Br and PRO with HDAC6 was calculated to be −8.4 kcal/mol and −8.1 kcal/mol (Figures 5C–F), respectively.

### PRO-Br promoted chaperone-mediated autophagy for pathological tau clearance

**FIGURE 6:**
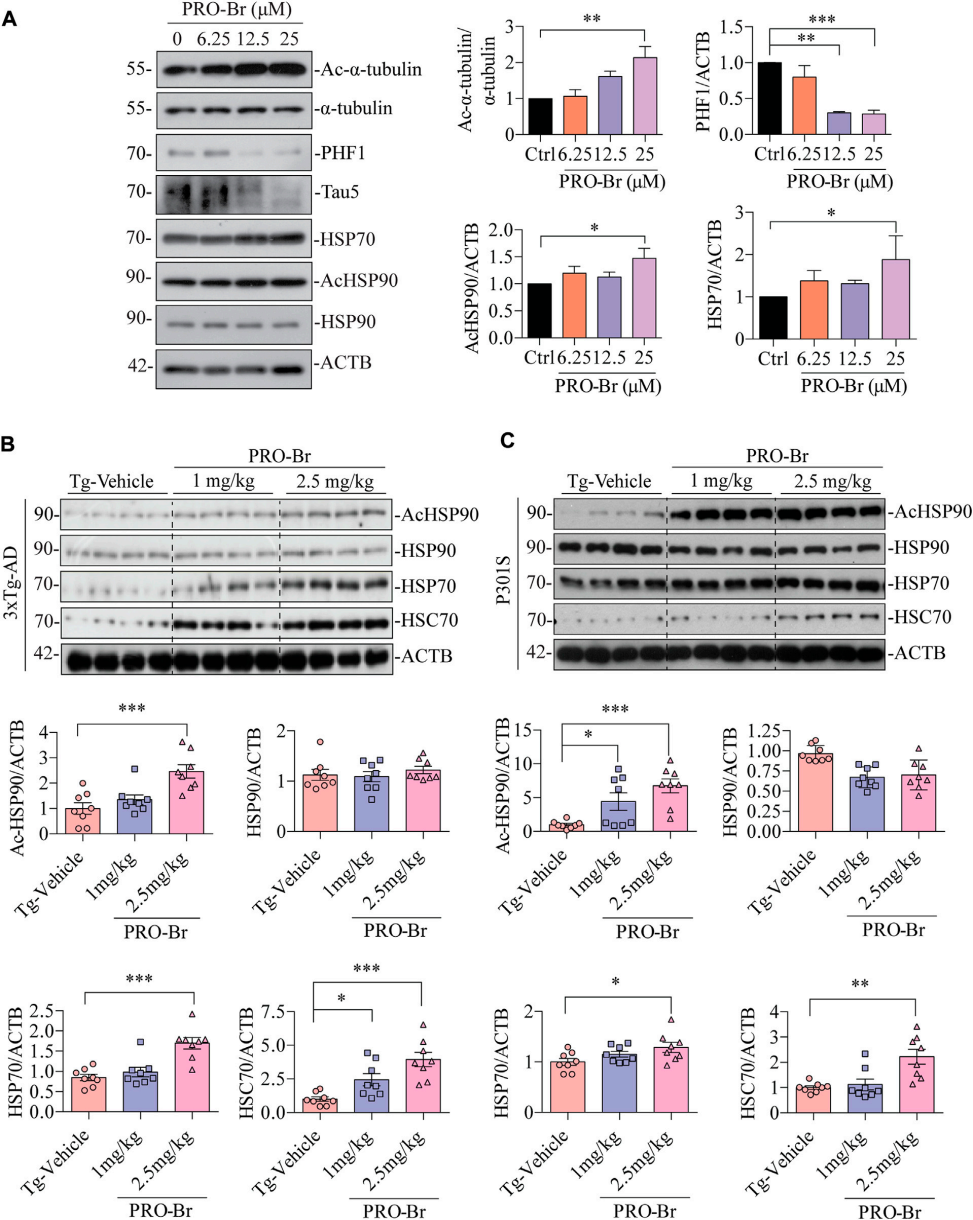
PRO-Br enhanced molecular chaperones for the degradation of PHF1. In SHSYP301L cells, **(A)** PRO-Br dose-dependently increased molecular chaperones. PRO-Br enhances molecule chaperones dose-dependently in **(B)** 3xTg-AD and **(C)** P301S mice models. Data was quantified using ImageJ software, *p < 0.05, **p < 0.01 and ***p < 0.001 vs. Tg-Vehicle (one-way ANOVA).

Autophagy-lysosomal pathway (ALP) and ubiquitin-proteasomal system (UPS) are two major mechanisms involved in the degradation of pathological tau, and these degradative pathways are known to be disrupted in AD (Orr and Oddo, 2013) (Thibaudeau et al., 2018). Several reports have linked defective proteolytic processes with AD. Hence, activation of cellular clearance mechanisms is regarded as a neuroprotective strategy for AD therapy. Recent findings from our group and others have demonstrated that activation of ALP or UPS caused a decline in pathological tau and improved cognitive abilities in various AD mice models (Zhu et al., 2022) (Silva et al., 2020). In our previous work, we have observed that PRO from *Corydalis yanhusuo* can reduce hyper-phosphorylated tau *via* the activation of the UPS and also rescued animal models of AD from cognitive impairment (Sreenivasmurthy et al., 2022). Hence, we analyzed whether PRO-Br promoted the clearance of hyperphosphorylated tau (PHF1) through ALP or UPS. Interestingly, co-treatment of PRO-Br with a proteasome inhibitor (MG132) or autophagy inhibitors (Wortmannin, Wort) or lysosome inhibitor (Chloroquine, CQ) did not block PRO-Br-mediated PHF1 degradation (Supplementary Figure S5A), indicating that other mechanism(s) were critical to the proteolytic cleavage of PRO-Br. Earlier studies have demonstrated an inverse relationship between expression of molecular chaperones and tau pathogenesis (Dou et al., 2003). Hence, we investigated whether PRO-Br increased the protein expression of molecular chaperones. Firstly, in tau over-expressed neuronal cells, PRO-Br treatment significantly and dose-dependently augmented the expression of HSP70, simultaneously reducing PHF1 and TAU5 levels (Figure 6A). Next, in 3xTg-AD and P301S mice, we demonstrated that PRO-Br treatment dose-dependently increased the heat shock proteins HSP70 and HSC70. Previous studies have demonstrated that inhibition of HDAC6 promotes acetylation of HSP90, which impairs the interaction of HSP90 with its client proteins (Scroggins et al., 2007). Interestingly, in both AD mice models, we observed that PRO-Br dose-dependently enhanced the acetylation of HSP90 (K294), indicating inhibition of HSP90 chaperonic activity (Figures 6B,C).

**FIGURE 7:**
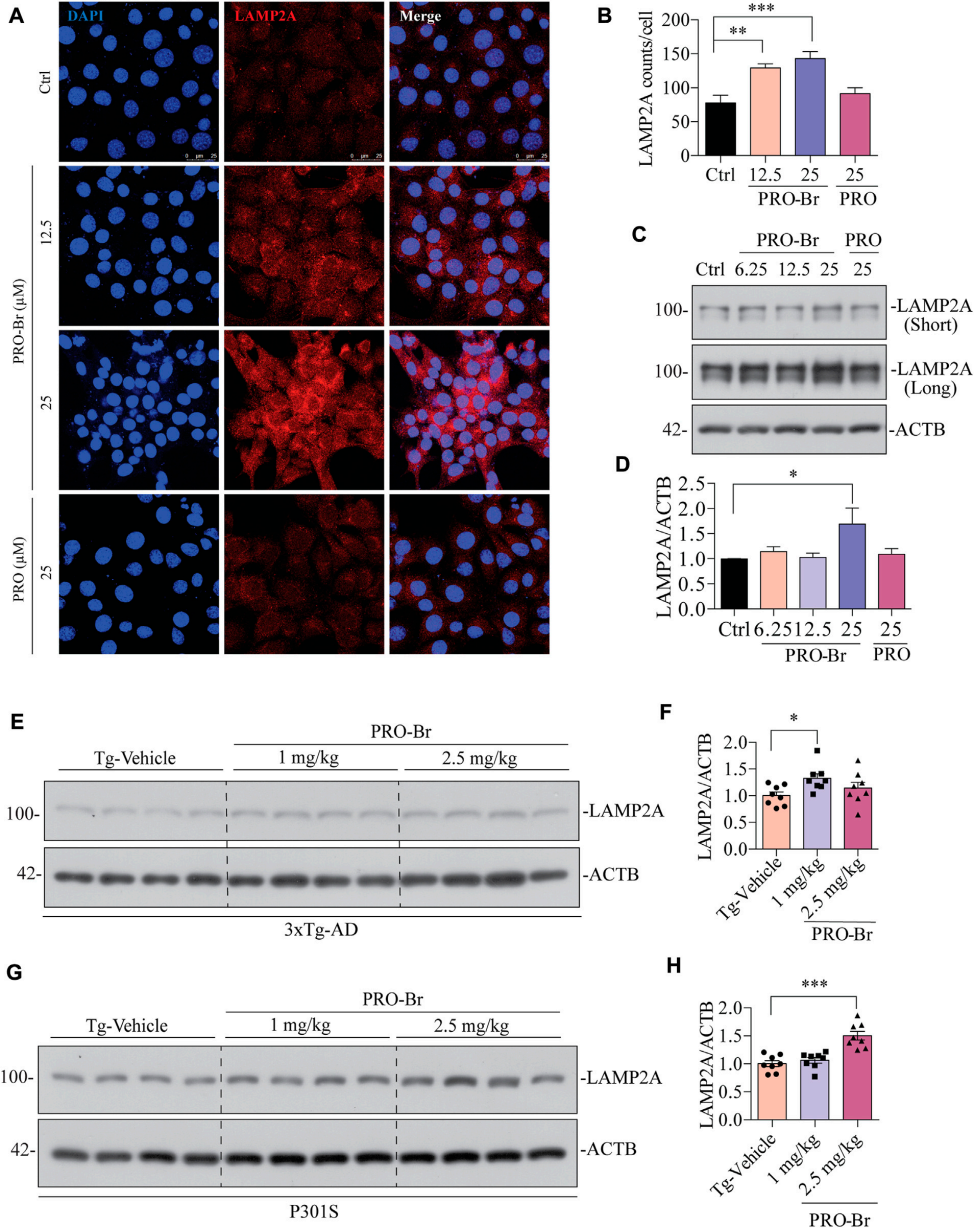
PRO-Br increased chaperone-mediated autophagy markers *in vitro* and *in vivo* **(A-B)** immunostaining, and **(C-D)** immunoblotting results show that PRO-Br treatment dose-dependently increases LAMP2A in HT22 cells. LAMP2A is shown in red and DAPI is shown in blue. Data are represented as mean ± SEM of three replicates. PRO-Br-mediated increase in expression of LAMP2A in **(E-F)** 3xTg-AD and **(G-H)** P301S mice models. Data quantification was performed by ImageJ software, *p < 0.05 and ***p < 0.001 vs. Tg-Vehicle (one-way ANOVA).

**FIGURE 8:**
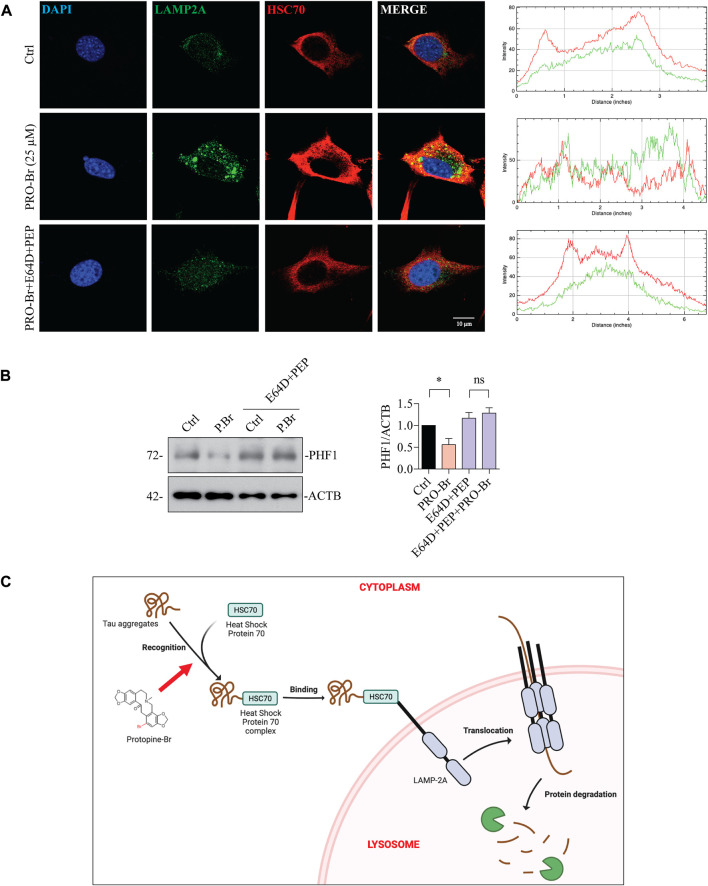
PRO-Br promotes the degradation of pathological tau *via* chaperone-mediated autophagy. In HT22 cells, **(A)** PRO-Br promotes the interaction between heat-shock protein cognate 71, HSC70 and lysosomal membrane protein, LAMP2A and its quantification. HSC70 is shown in red, LAMP2A is shown in green, DAPI is shown in blue, and the interaction is shown in yellow. In SHSYP301L cells **(B)** PRO-Br-mediated degradation of PHF1 was blocked in the presence of CMA/lysosomal protease inhibitors (E64D + PEP). Data was quantified using ImageJ software, *p < 0.05 and ns = not significant. Data are represented as mean ± SEM of three replicates. **(C)** Proposed graphical representation of the neuroprotective mechanism of PRO-Br. PRO-Br clears hyper-phosphorylated and aggregated tau *via* the activation of chaperone-mediated autophagy.

Next, we determined the possible effect of PRO-Br on chaperone-mediated autophagy (CMA), which is usually augmented as a response to cellular stressors. One of the well-studied markers for CMA activation is the enhanced expression of lysosome-associated membrane protein 2A (LAMP2A) (Kaushik and Cuervo, 2018) (Gong et al., 2018). Therefore, we evaluated the expression of LAMP2A in neuronal cells. In HT22 cells, immunocytochemical analysis showed that PRO-Br treatment distinctly enhanced the expression of LAMP2A in a dose-dependent manner, whereas PRO treatment did not enhance LAMP2A protein expression (Figures 7A,B). Additionally, immunoblotting results demonstrated that PRO-Br significantly enhanced LAMP2A expression in a dose-dependent manner compared to PRO (Figures 7C,D). Next, N2A cells were treated with PRO-Br, and we observed an increased expression of LAMP2A and HSC70, a heat shock cognate 71 protein, compared to Trehalose (Treh), a positive control (Supplementary Figure S6A–D). The protein expression of LAMP2A was dose-dependently increased in 3xTg-AD (Figures 7E,F), and P301S tau mice models (Figures 7G,H). In CMA, HSC70 and LAMP2A interact together for promoting the degradation of hyperphosphorylated tau *via* the lysosomes. Therefore, in this study we determined the interaction of PRO-Br-mediated HSC70-LAMP2A complex formation. In HT22 cells, immunofluorescence results demonstrated that PRO-Br markedly enhanced the interaction between HSC70 and LAMP2A (Figure 8A). Furthermore, to determine the degradation of hyperphosphorylated tau (PHF1), PRO-Br-treated cells were co-incubated with CMA/lysosomal protease inhibitors, E64D and pepstatin (PEP). Immunoblotting results from tau-overexpressed cells demonstrated that co-treatment of PRO-Br and CMA inhibitors significantly blocked the degradation of PHF1 levels, compared to PRO-Br treatment alone (Figure 8B). Collectively, our results indicate that PRO-Br can promote the degradation of pathological tau *via* activating CMA, *in vitro* and *in vivo*.

## Discussion

AD pathology remains elusive, which might explain the failures in several clinical trials (Mehta et al., 2017). In the present study, PRO-Br, a derivative of PRO has been assessed for its anti-AD efficacy in AD models. *In vivo* study demonstrated that PRO-Br can efficiently pass through the BBB and attain higher concentration compared to PRO. In 3xTg-AD and P301S tau mice models, PRO-Br significantly mitigated memory and cognitive impairment and promoted the clearance of pathological tau load. Our *in vitro* study demonstrated that PRO-Br is a better HDAC6 inhibitor, compared to the parent compound. Also, PRO-Br enhanced the expression of HSC70 and LAMP2A in AD models. Furthermore, PRO-Br mediated the interaction between HSC70 and LAMP2A and facilitated the degradation of pathological tau. The above collective results demonstrate the fundamental mechanism of PRO-Br-mediated pathological tau clearance in AD models is *via* the activation of chaperone-mediated autophagy.

Previously published research has established that PRO, the parent natural compound of PRO-Br, can significantly promote the clearance of pathologically phosphorylated tau and protect AD mouse models from severe memory and cognitive dysfunction. Although previous studies have established that PRO has anti-inflammatory, antioxidant, antinociceptive, and anti-acetylcholinesterase activity, PRO’s oral bioavailability is low. There has been no attempt to modify or enhance the biological activity of PRO structurally. To improve PRO’s activity, we conducted a structure-activity relationship study and synthesized several analogs of PRO, which were then tested to determine which had the strongest anti-AD activity.

PRO was modified at the C13 position to obtain 3a (methyl), 3b (ethyl), and 3c (benzyl) derivatives. Chemical reactions were performed on the carbonyl carbon to obtain 5a (acryl), 5b (methyl), 5c (cyclopentyl) and 5d (cyclohexyl) derivatives of PRO. Further reactions were performed on the C12 position to obtain 7b (acyl), 7c (benzyl), 7d (methoxy benzyl), and 7e (CO2Et). Bromine group substitution at the C12 position on PRO yielded the brominated derivative (PRO-Br). It is noteworthy that modification of PRO in the C12 position alone can enhance the efficacy since C12 in an important active C in PRO. According to our results, we have seen that PRO-Br can bind to HDAC6 and inhibit its activity, and promote chaperone-mediated autophagy (CMA) to reduce PHF1-tau. It is possible that some of the synthesized PRO derivatives may not bind to HDAC6 and inhibit its function, and also the compounds might not enhance CMA activity. Moreover, after structural modification, the compounds may have lost the tau-reducing activity of parent compound.

Previously published research indicated that small molecules (Choi et al., 2020) could promote the clearance of hyperphosphorylated tau, but they did not evaluate the activity of small molecules on non-pathological tau. In this study, we determined that PRO-Br promotes the degradation of hyperphosphorylated tau, specifically in sarkosyl-insoluble fractions, in 3xTg-AD and P301S mice models. Additionally, immunohistochemical brain slices of 3xTg-AD mice treated with PRO-Br demonstrated a dose-dependent reduction in AT8-positive cells. PRO-Br treatment decreased the number of hyperphosphorylated tau-positive cells in the P301S mouse model, and also exhibited exceptional efficacy in reversing motor and memory impairments in 3xTg-AD and P301S mouse models. Recently, research has shown that HDAC6 plays a crucial role in compromising memory and cognitive functions in AD rodent models (Choi et al., 2020). Besides, recent studies have demonstrated that CM695 can inhibit HDAC6 (Cuadrado-Tejedor et al., 2019). Nevertheless, direct modulation of HDAC6 activity by these compounds is not well studied. Our current study demonstrates that PRO-Br binds to HDAC6 and further inhibits its activity, thereby promoting tau degradation in AD models.

HDAC6 can deacetylate HSP90 and enhance the binding of tau oligomers to deacetylated HSP90 (Weickert et al., 2020). PRO-Br inhibits HDAC6 activity and promotes HSP90 acetylation, thereby recruiting HSC70 for tau degradation *via* chaperone-mediated autophagy (CMA) in AD models (Figure 8C). Thus, our findings are consistent with those of these other studies, which have demonstrated that acetylation of HSPs is required for tau to be recruited for the chaperone-mediated clearance of pathological tau (Petrucelli et al., 2004).

From both the 3xTg-AD and P301S mouse models, we conclude that PRO-Br can efficiently cross the blood-brain barrier and promote the degradation of insoluble pathological tau *via* the chaperone-mediated autophagy. PRO-Br binds to and inhibits the activity of HDAC6, thereby promoting the recruitment of molecular chaperones for tau degradation. PRO-Br improves motor and cognitive function in models of Alzheimer’s disease. Taken together, our compelling findings suggest that PRO-Br administration may be a viable anti-AD therapeutic strategy.

## Data Availability

The original contributions presented in the study are included in the article/Supplementary Material, further inquiries can be directed to the corresponding authors.

## Supplementary material

Supplementary Figure S1 Pharmacokinetic parameters of PRO-Br. Table illustrating the pharmacokinetic parameters of PRO-Br (10 mg/kg) in **(A)** brain, and **(B)** plasma samples.

Supplementary Figure S2 Synthesis of protopine derivatives. Several chemical reactions were performed by targeting the C13 position to obtain 3a (methyl), 3b (diallyl), and 3c (dibenzyl), 4 (hydroxy), 5b (methyl), and 5c (cyclopentyl) derivatives of protopine. Chemical reaction with N-bromosuccinimide was performed on the C12 position to obtain 7a (Bromo).

Supplementary Figure S3 Physical and chemical characterization of PRO-Br. **(A,B)** NMR and **(C)** LC-MS analysis. **(D)** Decoded NMR spectra.

Supplementary Figure S4 PRO-Br can improve dendritic spine density in the 3xTg-AD mice model. Golgi staining images show that **(A)** PRO-Br increased dendritic spine density in female 3xTg-AD mice. **(B)** The bar graph shows that PRO-Br increased all the types of spines (thin, stubby, and mushroom-like) dose-dependently. N = 8, bar graph represents mean ± SEM, **p* < 0.05; ****p* < 0.001 (Tg-PRO vs Tg-Vehicle).

Supplementary Figure S5 PRO-Br-mediated tau degradation is independent of ALP and UPS in vitro. **(A)** Immunoblotting results show co-treatment of PRO-Br with autophagy inhibitors, chloroquine (CQ), wortmannin (Wort), and proteasomal inhibitor (MG132) in SHSYP301L cells. The expression of phosphorylated tau was analyzed with a specific monoclonal antibody (PHF1). Data are represented as mean ± SEM of 3 replicates. Data were quantified using Image J software, **p* < 0.05 and ###*p* < 0.001.

Supplementary Figure S6 PRO-Br promoted chaperone-mediated autophagy in-vitro. In N2A cells, **(A,B)** immunofluorescence images show that PRO-Br enhances LAMP2A expression dose-dependently. LAMP2A (green) and DAPI (blue). **(C,D)** Immunoblotting results show that PRO-Br mediated the increase in LAMP2A and HSC70 expression in a dose-dependent manner. Data are represented as mean ± SEM of 3 replicates. Data were quantified using Image J software, **p* < 0.05 and ***p* < 0.01.
