# Supplementary figures and images for: Bromo-protopine, a novel protopine derivative, alleviates tau pathology by activating chaperone-mediated autophagy for Alzheimer’s disease therapy
Source: Front Mol Biosci. 2022 Oct 26;9:1030534. doi: 10.3389/fmolb.2022.1030534 (PMC9643865; doi:10.3389/fmolb.2022.1030534)

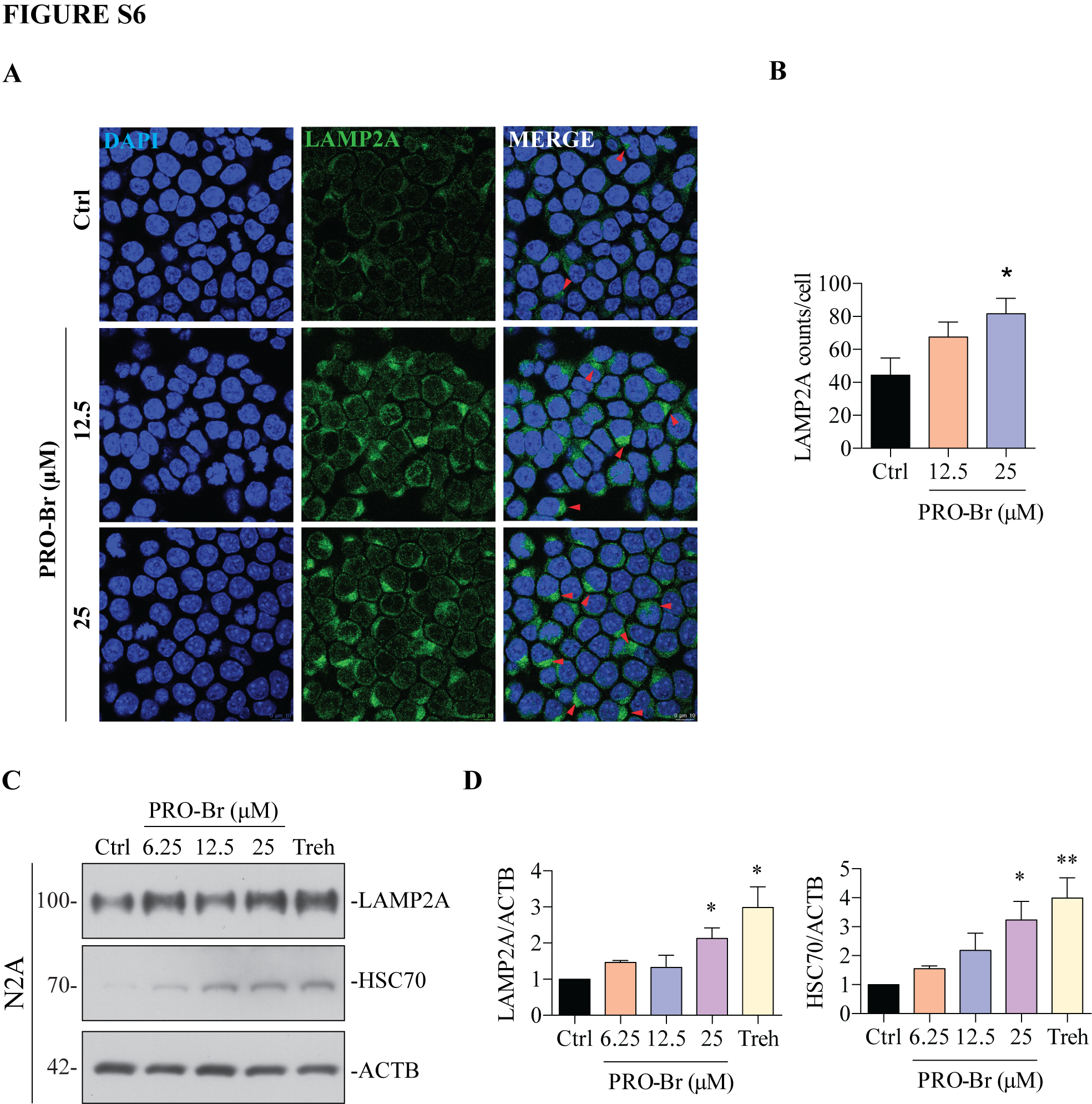

Supplement: Supplementary file 1 [file Image6.TIF]

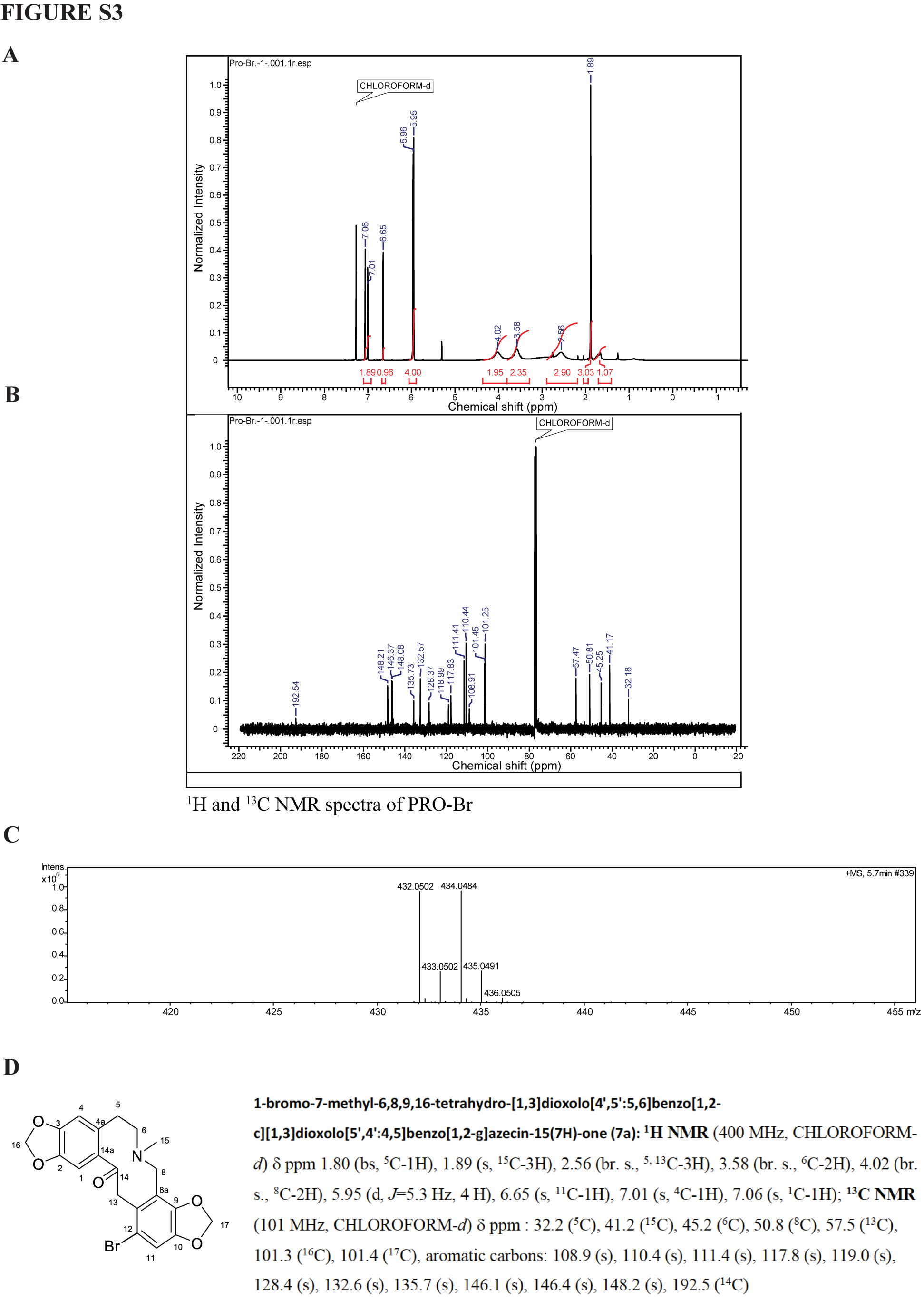

Supplement: Supplementary file 2 [file Image3.TIF]

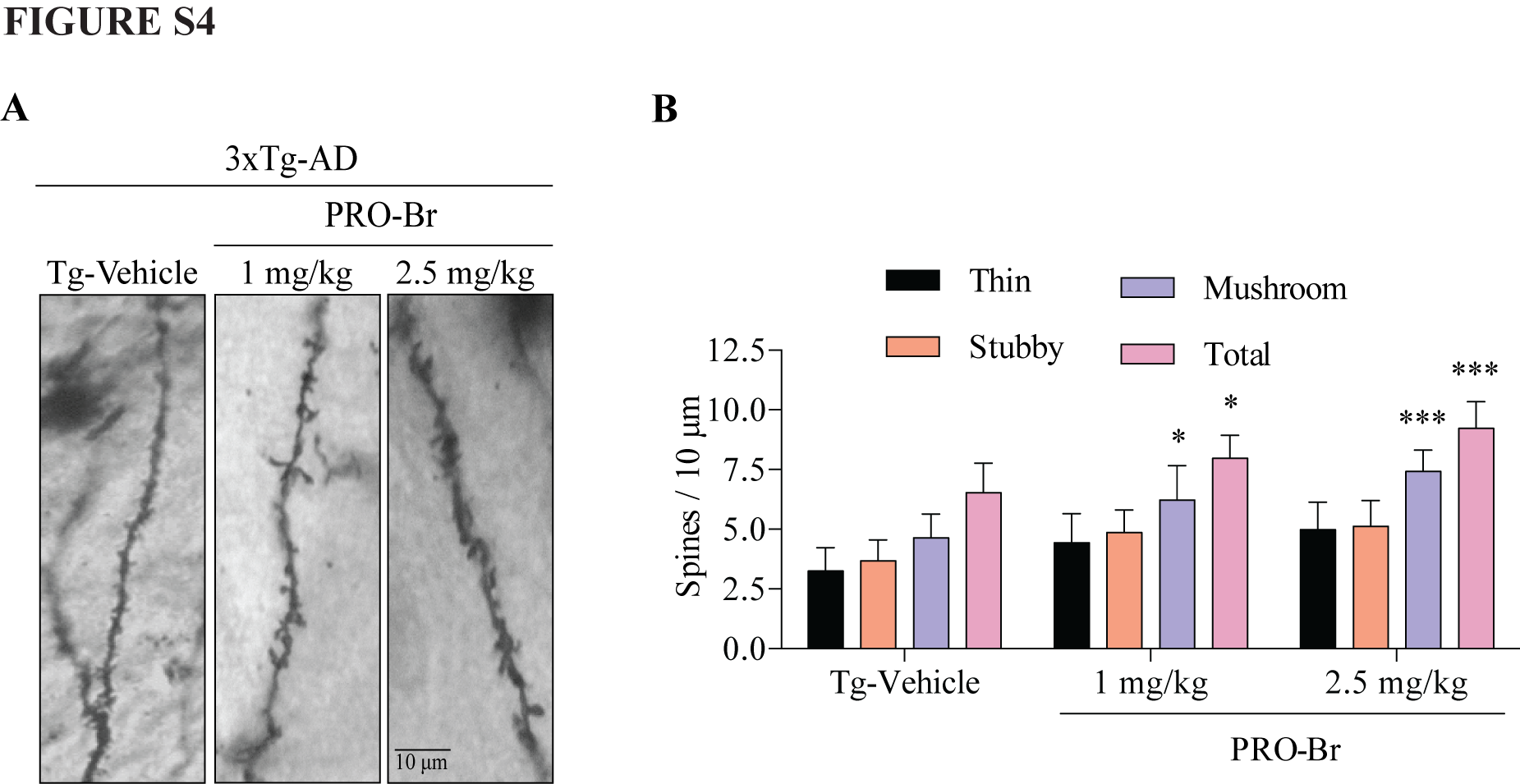

Supplement: Supplementary file 3 [file Image4.TIF]

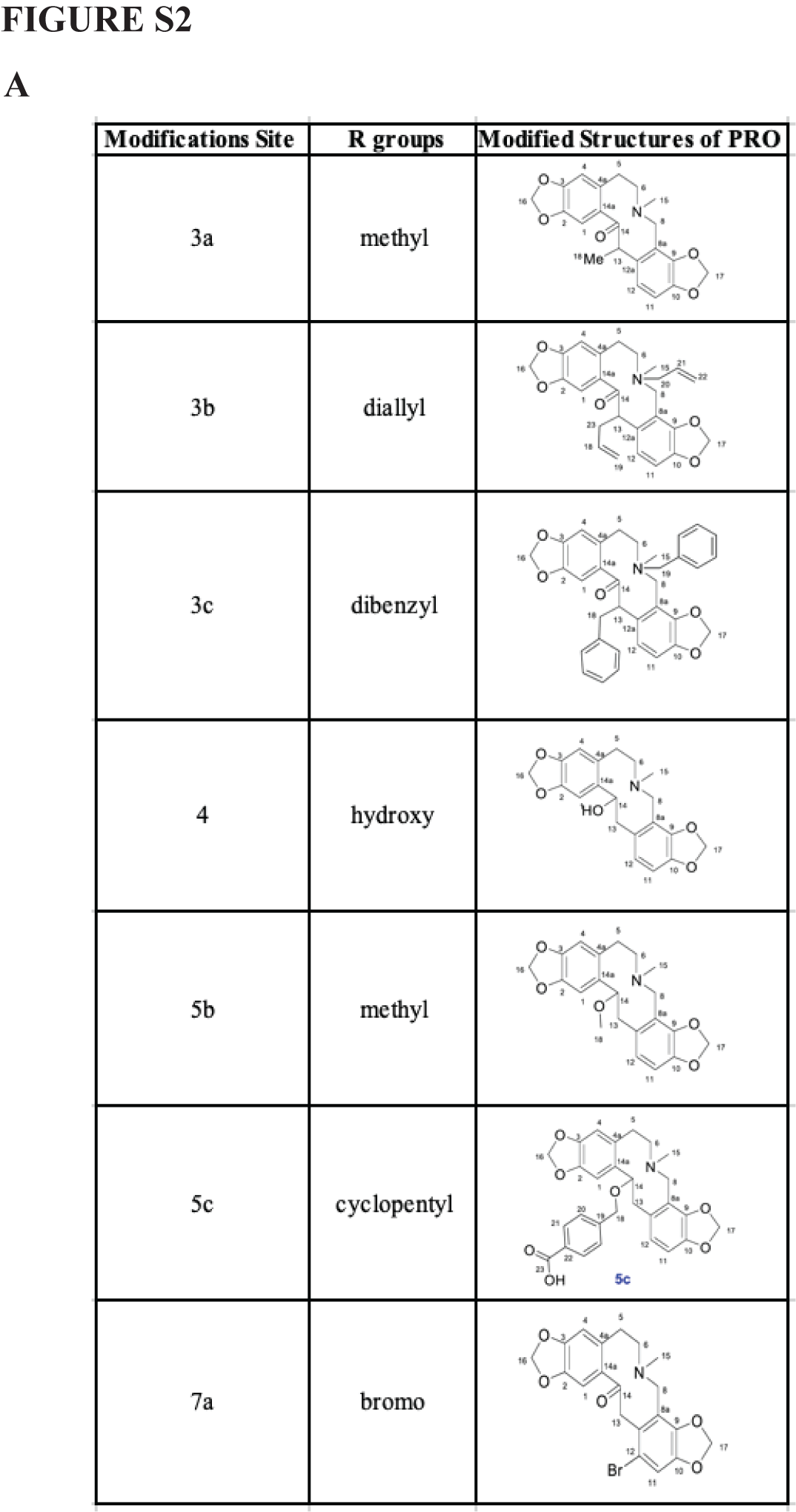

Supplement: Supplementary file 4 [file Image2.TIF]

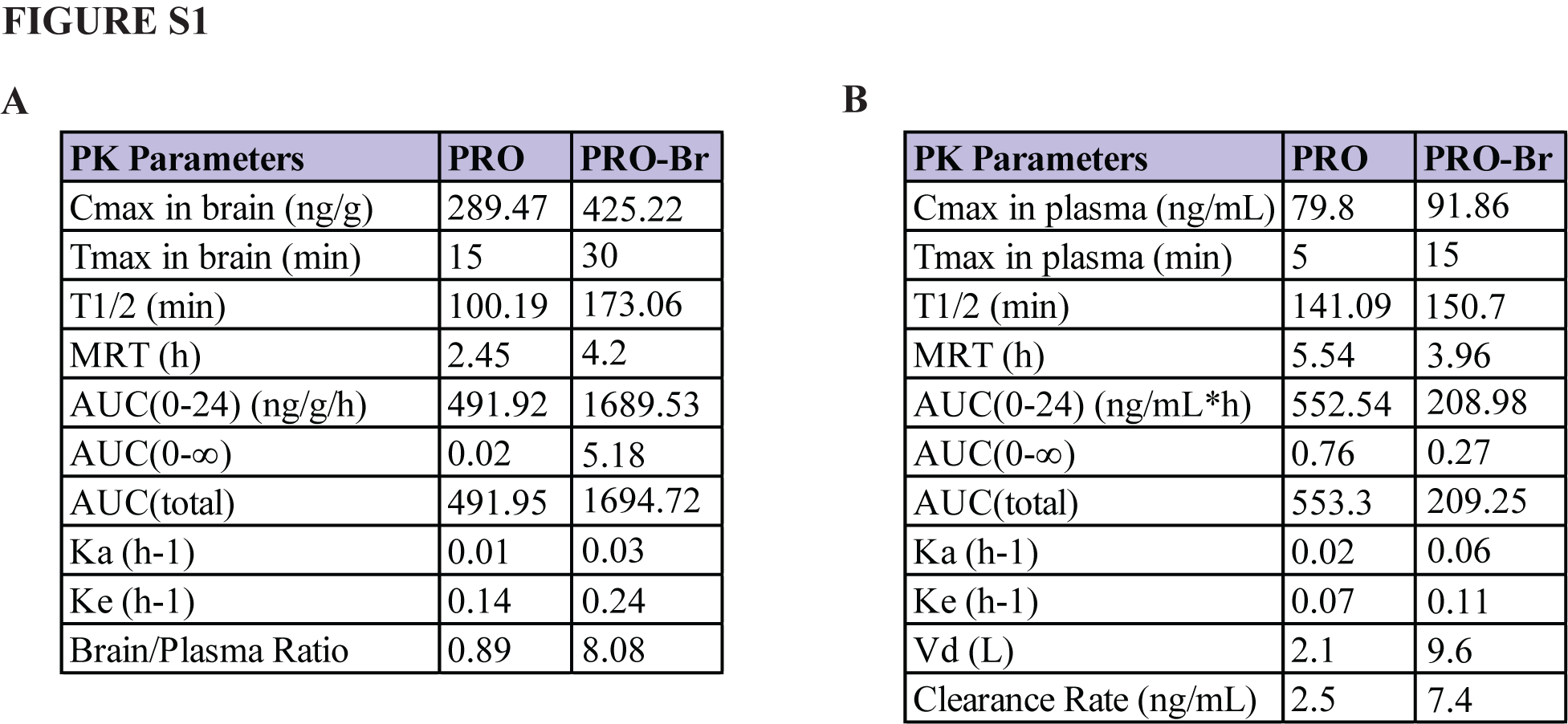

Supplement: Supplementary file 5 [file Image1.TIF]

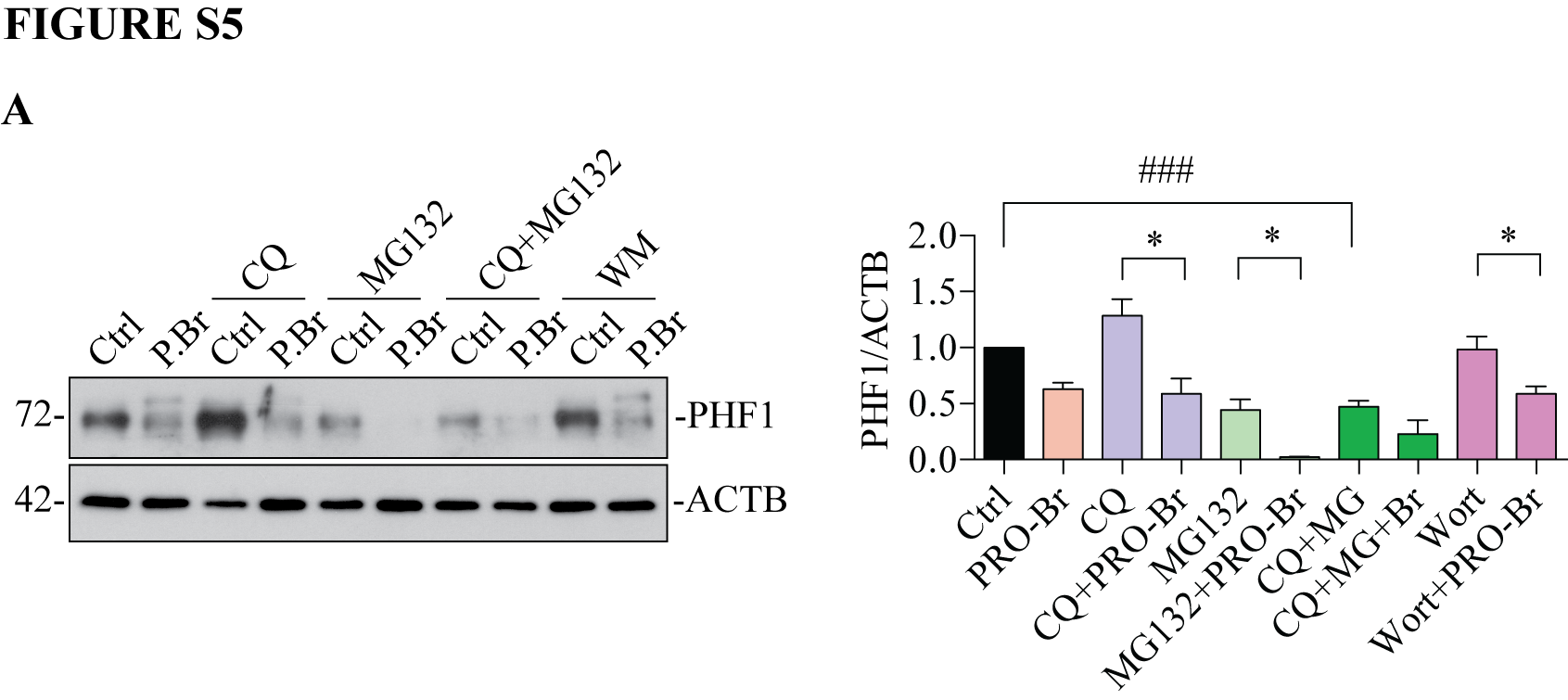

Supplement: Supplementary file 6 [file Image5.TIF]
